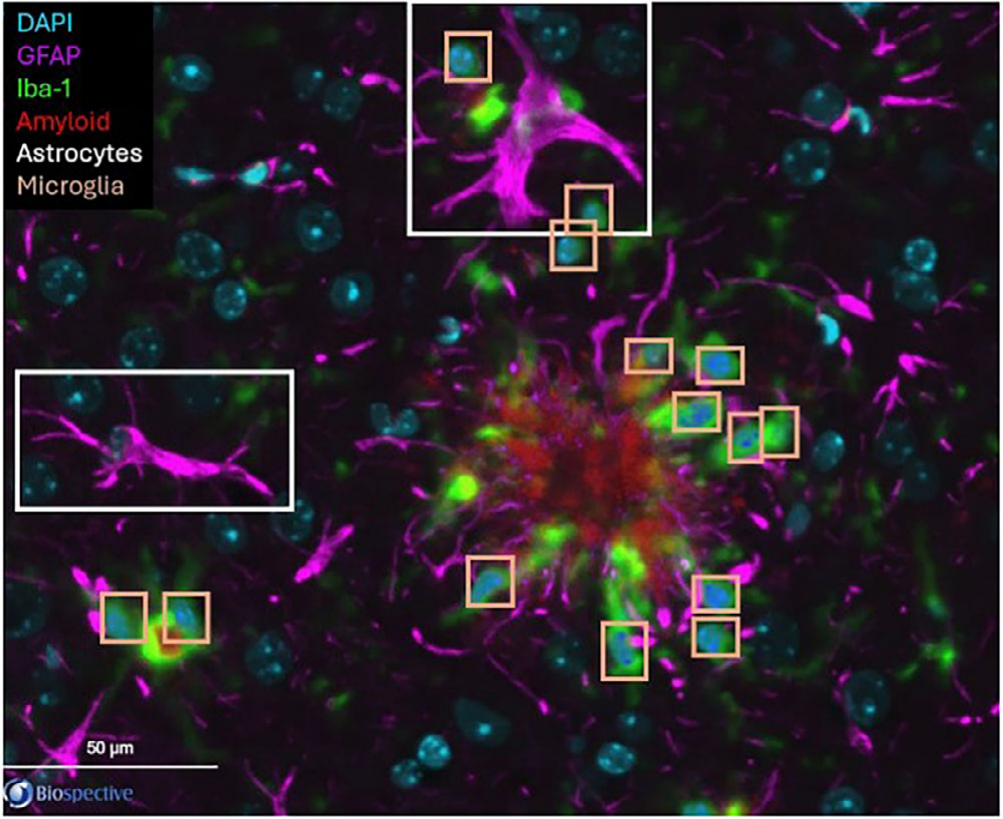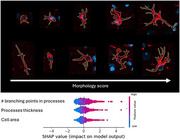# Morphological analysis of astrocytes and microglia in the amyloid‐beta plaque microenvironment of a mouse model of Alzheimer's disease

**DOI:** 10.1002/alz70855_099341

**Published:** 2025-12-27

**Authors:** Laurent Potvin‐Trottier, Robin Guay‐Lord, Lionel Breuillaud, Simone P Zehntner, Elodie Brison, Barry J Bedell

**Affiliations:** ^1^ Biospective Inc, Montreal, QC, Canada; ^2^ Biospective Inc., Montreal, QC, Canada

## Abstract

**Background:**

Astrocytes and microglia are thought to play a key role in many neurodegenerative diseases, including Alzheimer's disease. Understanding the specific subtypes, roles, and interactions of astrocytes and microglia is important to elucidate disease mechanisms and to identify and assess therapeutic targets. This study aimed to evaluate the spatiotemporal dynamics of astrogliosis and microgliosis in the amyloid‐beta plaque microenvironment in an APP/PS1 transgenic mouse model of Alzheimer's disease.

**Method:**

We previously developed an automated method to analyze the plaque microenvironment in multiplex immunofluorescence tissue sections, quantifying stain density and characterizing microglia morphology. We have extended this work and implemented a deep learning‐based approach to identify, count, and localize astrocytes (Figure 1). Astrocyte morphology was assessed using an explainable machine‐learning (ML) model to distinguish cells with hypertrophic morphology, indicative of reactivity. We also developed a ML model to distinguish vascular from non‐vascular amyloid‐beta plaques. We leveraged these methods to measure spatiotemporal cellular changes in the plaque microenvironment in tissue sections stained for Aβ, Iba‐1, GFAP, and DAPI from mice at 6, 9, and 12 months‐of‐age.

**Result:**

Our model classified reactive astrocytes based on distinctive morphological features, such as thicker, more branched processes (Figure 2). A score based on these morphological features provided a sensitive measure of disease progression. The mean astrocyte hypertrophy score showed changes with greater statistical significance than GFAP stain density. To study the spatiotemporal evolution of the plaque microenvironment, we quantified the density of reactive cells in the microenvironment as a function of plaque size. We found that microglia progressively accumulate as a function of plaque size in the immediate vicinity. In contrast, hypertrophic astrocytes accumulate more distantly than microglia and are progressively excluded from the larger plaques. Finally, we found that the microenvironment of vascular plaques had much less neuroinflammation than the non‐vascular plaques.

**Conclusion:**

Assessment of morphological characteristics can provide additional information about the astrocytic phenotype. These features may provide sensitive measures for preclinical assessment of putative disease‐modifying therapeutic agents in rodent models of neurodegenerative diseases.